# Polyvinyl Alcohol/Polyaniline/Carboxylated Graphene Oxide Nanocomposites for Coating Protection of Cast Iron in Simulated Seawater

**DOI:** 10.3390/polym14091791

**Published:** 2022-04-27

**Authors:** Noha A. Elessawy, Marwa H. Gouda, Mohamed Elnouby, Nahla A. Taha, M. Elsayed Youssef, Diogo M. F. Santos

**Affiliations:** 1 Computer Based Engineering Applications Department, Informatics Research Institute IRI, City of Scientific Research and Technological Applications (SRTA-City), Alexandria 21934, Egypt; elsayed168@gmail.com; 2 Polymer Materials Research Department, Advanced Technology and New Materials Research Institute, City of Scientific Research and Technological Applications (SRTA-City), Alexandria 21934, Egypt; 3 Nanomaterials and Composites Research Department, Advanced Technology and New Materials Research Institute, City of Scientific Research and Technological Applications (SRTA-City), Alexandria 21934, Egypt; mnano2050@yahoo.com; 4 Modelling and Simulation Research Department, Advanced Technology and New Materials Research Institute (ATNMRI), City of Scientific Research and Technological Applications (SRTA-City), Alexandria 21934, Egypt; nahlataha_1982@yahoo.com; 5 Center of Physics and Engineering of Advanced Materials, Laboratory for Physics of Materials and Emerging Technologies, Chemical Engineering Department, Instituto Superior Técnico, Universidade de Lisboa, 1049-001 Lisbon, Portugal; diogosantos@tecnico.ulisboa.pt

**Keywords:** polyvinyl alcohol, polyaniline, carboxylated graphene, saline environment, corrosion inhibition

## Abstract

In our daily lives and product manufacturing, metal corrosion causes significant economic losses. Numerous polymeric composite coatings have been shown to be resistant to harsh environments, such as those found in marine environments. In this study, a composite of polyvinyl alcohol/polyaniline blend loaded with carboxylated graphene was explored in the search for long-lasting coatings to resist electrochemical deterioration of cast iron in desalination systems of saltwater. Polyvinyl alcohol/polyaniline/carboxylated graphene oxide nanocomposite was spin-coated onto cast iron samples. Electrochemical impedance spectroscopy (EIS) and electrochemical DC corrosion testing with a three-electrode system were used to study corrosion resistance in uncoated and coated cast iron samples. The results exhibit effective corrosion protection properties. The EIS data indicated better capacitance and higher impedance values for coated samples than bare metal, depicting enhanced corrosion resistance against the saline environment. Tafel analysis confirmed a significant decrease in the corrosion rate of the PVA/PANI/GO-COOH coated sample.

## 1. Introduction

Seawater desalination develops rapidly, as it can solve water scarcity efficiently, whereas desalination, cooling, and full utilization of seawater are all effective ways to meet human water needs. However, corrosion problems for most metals and alloys in seawater desalination systems are more severe than in regular water because seawater is rich in natural electrolytes and highly corrosive substances, which cause general and localized corrosion. Both types of corrosion harm the service life of seawater desalination equipment and the safe operation of the system. Corrosion in seawater also causes high economic costs, such as lost production, product loss, efficiency loss, and product contamination. Even worse, it may cause catastrophic mishaps, such as harmful chemical leaks, resulting in pollution and putting people’s health at risk [1]. As a result, it is critical to pay attention to corrosion inhibition for metals in a saline environment by investigating corrosion behavior and taking appropriate anti-corrosion measures to use marine resources efficiently and actively respond to sustainable development.

Standard coating systems for metal protection incorporate more than one type of coating system. However, protective coatings [2], corrosion inhibitors [3], nanocomposites [4], and electrochemical protection [5,6] are now the most widely utilized ways of improving corrosion resistance. Furthermore, various coating systems have been proposed by many researchers that effectively prevent metals from corrosion. Still, research related to non-toxic and cost-effective coating system that exhibits excellent corrosion resistance behavior is limited.

Furthermore, in the corrosion protection of metals, polymer-based coatings are widely used since they possess strong binding capability with the metal surface that acts as a barrier. Through their functional groups, they form complexes with metal ions. These complexes occupy a large surface area on the metal surface and act as a blanket to the surface, protecting the metal from corrosive agents present in the solution [2,4]. However, the presence of micropores and microcracks generated during the coating formation leads to the failure of the coating due to the permeation of corrosive ions. According to recent research, loading the polymeric matrix with conductive polymers and nanoparticles can increase the active surface area of the coating, improve its electrostatic conductivity, and stimulate the creation of a passivation layer at the interface between metal and polymer [4,7,8].

Polyvinyl alcohol (PVA) is a non-toxic and water-soluble synthetic polymer that shows film-forming characteristics. PVA is also used as an anti-corrosion coating because it acts as a high oxygen barrier and adheres firmly to the metal surface [9,10,11]. On the other hand, coatings containing conducting polymers such as polyaniline (PANI) can protect pinholes and defects due to their passivating ability [7,8]. The PANI protection mechanism is electronic in nature, similar to the mechanism seen in metals. Furthermore, there are various reports about the improvement of coatings performance using nanoparticles as reinforcement, such as graphene (G) and graphene oxide (GO, the oxide of graphene) [4,12,13,14]. GO has several unique properties, such as good dispersion capability, and some of graphene’s basic characteristics, such as a large specific surface area, and excellent electrical and thermal conductivity [4,14]. However, by combining GO with PVA, the oxygen functional groups present in GO form a strong molecular interaction with PVA [15,16] and enhance the strength of the coating layer.

Therefore, the development of hybrid materials with multiple combinations of polymers and GO is gaining attraction and can bring stability to the coating. It has been demonstrated that PVA has low water absorption behavior. It behaves as a corrosion inhibitor by forming complexes and covering surfaces to protect the metallic material from corrosion [9,10,17]. The dielectric properties of PANI indicate its capability to provide reasonably good corrosion protection [18]. Additionally, GO, which was used as reinforcement in both coatings, has also demonstrated significant anti-corrosion properties due to its hydrophobic nature, as a result of its non-polar covalent double bond [4,14]. However, GO cannot act as an active corrosion protection coating for long-term applications. The pores-deformations formed on the GO coating may increase the corrosion rate compared to the uncoated substrate [19].

To the best of our knowledge, there were no reports in the literature dealing with the direct deposit of PVA/PANI/GO-COOH nanocomposite coating on cast iron from an aqueous solution. The present study deals with the preparation of PVA/PANI blended with carboxylated graphene oxide (GO-COOH) prepared from upcycled plastic waste to form a novel nanocomposite coating. The characteristics of different types of prepared PVA, PVA/PANI, PVA/GO-COOH, and PVA/PANI/GO-COOH nanocomposite coatings were explored using FTIR analysis. In addition, these coatings were characterized by using weight loss measurements, potentiodynamic polarization studies, and electrochemical impedance analysis. Finally, the possibility of utilizing the PVA/PANI/GO-COOH nanocomposite coatings for corrosion protection of cast iron in an aqueous 3.5 wt.% NaCl solution was examined and optimized using the response surface methodology model. An excellent corrosion-resistant coating is expected by reducing the pores, with each constituent of the coating being believed to improve corrosion resistance. Furthermore, its cost of production is low, and large-scale implementation in commercial applications is comparatively convenient.

## 2. Materials and Methods

### 2.1. Materials

PVA (MW 89,000–98,000, +99% hydrolyzed), aniline monomer, *N*-methyl-2-pyrrolidone (NMP), ammonium persulfate (APS), HCl, methanol, benzimidazole (98%), sodium chloride, sodium hydroxide, chloroacetic acid (ClCH_2_COOH, purum ≥ 97.0%) were purchased from Sigma-Aldrich (St. Louis, MI, USA).

The chemical composition of the cast iron sample is shown in Table 1. Cast iron specimens of dimensions 3.7 × 1.2 × 0.2 cm^3^ were polished to a mirror finish, degreased with trichloroethylene, and used for weight loss and surface examination studies.

### 2.2. Preparation of Composite Coatings

#### 2.2.1. Preparation of Carboxylated Graphene Oxide (GO-COOH)

0.1 g of reduced GO prepared from plastic waste using the previously mentioned procedure [20,21] was subjected to 1 h sonication. The resultant suspension (50 mL) was mixed with NaOH (0.6 g) and chloroacetic acid ClCH_2_COOH (0.5 g). The mixture was sonicated for 2 h to convert the –OH groups of GO into –COOH via conjugation of acetic acid moieties to obtain GO-COOH [22]. The resulting GO-COOH solution was purified by repeated rinsing with deionized water and filtrations until the product was well dispersed in water.

#### 2.2.2. Preparation of PANI

For the polymerizations in the liquid state, aniline (0.2 M) in 100 mL 1 M HCl was oxidized with ammonium peroxydisulfate (0.25 M) in 0.1 M HCl. Solutions of a monomer and an oxidant were mixed after pre-cooling at 0 °C to start the oxidation. The appearance of green color indicated that the polymerization was completed. The solution was washed several times with acetone to remove the unreacted monomer and then dried to obtain the required PANI powder.

#### 2.2.3. Preparation of PVA Solution

10 wt.% PVA was dissolved in deionized water at 90 °C for 2 h until a completely clear solution was obtained.

#### 2.2.4. Preparation of PVA/PANI Blended GO-COOH Nanocomposite Coating

**Figure 1:**
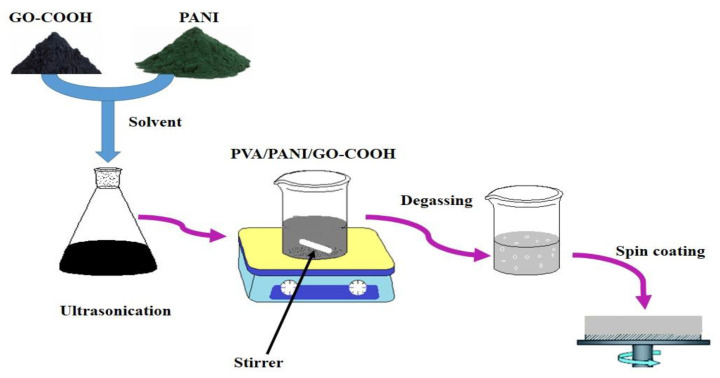
Schematic illustration of preparation process of the PVA/PANI blended GO-COOH nanocomposite coatings.

In the next step, the specific amount of PANI and GO-COOH were dissolved in 5 mL of NMP and kept on a sonication for 1 h to disperse, then added in PVA solution and mixed for 2 h. The PVA/PANI/GO-COOH nanocomposite composition was set as 98/0.5/1.5 wt.%. The complete process is schematically described in Figure 1.

For coating the cast iron sample surfaces, solutions covered the surface using POLOS 300 Advanced PTFE spin coater. For comparison, four coatings were investigated: PVA, PVA/PANI, PVA/GO-COOH, and PVA/PANI/GO-COOH nanocomposite coatings.

### 2.3. Characterization

Fourier transform infrared spectroscopy (FTIR) measurements of GO-COOH, PANI, PVA, and PVA/PANI/GO-COOH nanocomposites were obtained on a Nicolet 6700 spectrometer (Thermo Fisher Scientific, Waltham, MA, USA) at room temperature in the 4000–400 cm^−1^ range.

Weight loss experiments were conducted in 3.5 wt.% NaCl for 7 days at 25 °C. After the immersion period, the specimens were cleaned and reweighed to determine the corrosion rate [23]. Triple experiments were performed in each case, and the mean value of the weight loss is reported. Weight loss allows calculating the mean corrosion rate, as expressed in mg cm^−2^ h^−1^. The resulting quantity, corrosion rate (ω_corr_) is thereby the fundamental measurement of corrosion. ω_corr_ can be determined either by chemical analysis of dissolved metal in solution or by gravimetric method measuring the weight of specimen before and after exposure in the aggressive solution applying the following equation:

ω_corr_ = (*m_i_* − *m_f_*)/(*S.t*) (1)

where *m_i_*, *m_f_*, *S*, and *t* denote initial weight, final weight, specimen surface, and immersion time, respectively. 

The inhibition efficiency, ηω%, was determined as follows:

ηω% = (ω^0^_corr_ − ω_corr_)/ω^0^_corr_ × 100 (2)

where ω^0^_corr_ and ω_corr_ are the corrosion rates in the absence and presence of inhibition coating, respectively.

Tafel polarization, linear polarization resistance, and electrochemical impedance spectroscopy experiments have been performed for further corrosion investigation by using a three-electrode assembly cell having a cast iron working electrode, a graphite rod counter electrode, and a saturated calomel electrode as reference electrode, and using Potentiostat/Galvanostatic Metrohm Autolab (Utrecht, The Netherlands), AUT85664).

To test the stability of the prepared PVA/PANI/GO-COOH nanocomposite coating in the variable time interval with the solution temperature and saline solution concentration, the experimental data were evaluated and optimized using the response surface methodology (RSM) models. Data analysis and optimization were conducted using “Design Expert” 13.0.9.0 StatEase software. The results were analyzed using analysis of variance (ANOVA), standard residuals versus predicted values, and three-dimensional surface maps.

## 3. Results and Discussion

### 3.1. Characterization of Nanocomposite Coating Using FTIR Spectroscopy

**Figure 2:**
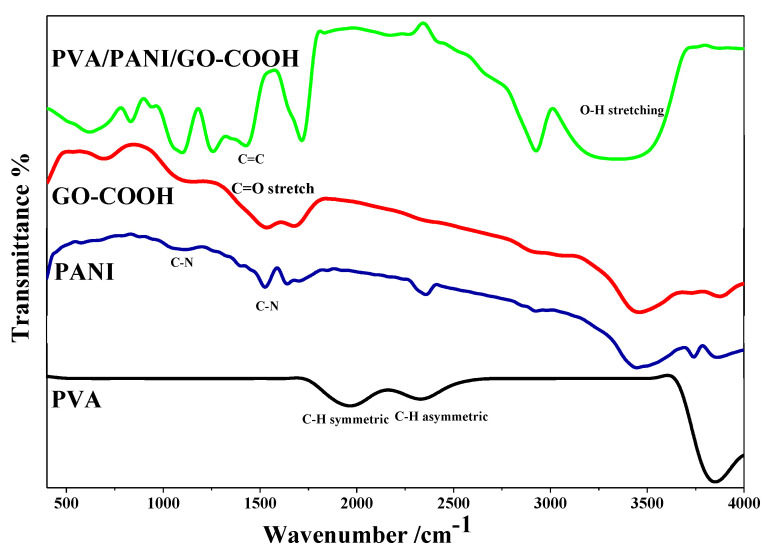
FTIR spectra of PVA, PANI, GO-COOH, PVA/PANI/GO-COOH composite coating.

Fourier transform infrared spectroscopy was used to investigate the functional groups of the prepared materials as shown in Figure 2. The FTIR spectrum of PVA consisted of a strong broad band at 3765 cm^−1^ corresponding to OH stretching vibration of hydroxyl groups of PVA. The band corresponding to C–H asymmetric stretching vibration occurred at 2428 cm^−1^, and C–H symmetric stretching vibration was observed at 2159 cm^−1^. However, the spectrum of PANI showed bands at 1580, 1460, 1300, and 1130 cm^−1^, which could be assigned to the C=C stretching of the quinoid ring, C=C stretching of the benzenoid ring, C–N stretching of the benzenoid unit, and C–N stretching of the quinoid unit, respectively [24]. FTIR spectrum of GO-COOH consisted of a strong broad band at 3446 cm^−1^, corresponding to O-H stretching, while the band at 1636 cm^−1^ corresponded to C=O stretch in carboxylic acids, and the band at 1393 cm^−1^ corresponded to O-H bend [25]. In the composite spectrum, esterification between the –OH group of PVA and –COOH of the GO-COOH appeared at 1716 cm^−1^, and the band at 1427 cm^−1^ could be assigned to the C=C stretching of the quinoid ring of PANI.

### 3.2. Corrosion Tests

#### 3.2.1. Corrosion Protection Performance of Prepared Coatings

**Figure 3:**
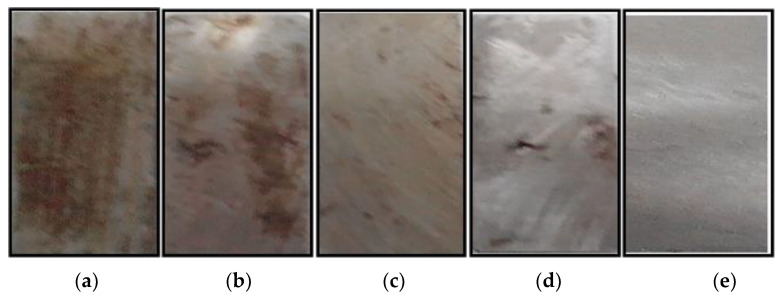
Optical images of cast iron coupons after 7 days of exposure to 3.5 wt.% NaCl (**a**) uncoated, (**b**) coated with PVA, (**c**) coated with PVA/PANI, (**d**) coated with PVA/GO-COOH, (**e**) coated with PVA/PANI/GO-COOH.

Prior to assessing the corrosion performance of composite coatings, the corrosion inhibition property of PVA/PANI/GO-COOH nanocomposite for cast iron coupon in 3.5 wt.% NaCl aqueous solution was examined as shown in Figure 3. After immersion in NaCl solution for 7 days, the surface of the cast iron coupon was covered with brown corrosion products (Figure 3a), while the sample coated with PVA/PANI/GO-COOH had no change in color (Figure 3e). Pure PVA coating, PVA blended PANI coating, and PVA doped with GO-COOH coating were also prepared for comparison. These results show that PVA/PANI/GO-COOH nanocomposite provided effective inhibition against corrosion. In addition, PVA, PVA/PANI, and PVA/GO-COOH composites also inhibited corrosion to some extent, as shown in Figure 3b–d. That may reveal that the interaction mechanism between the coating and the metal surface generally includes an adsorption phenomenon. The strength of the adsorption on a metallic surface depends largely on the electrostatic forces of attraction between the polar head of the polymer coating molecule with the iron atoms on the metal surface. The adsorption of the coating molecules on the metal surface is not only a precursor to surface adhesion, which forms a physical barrier to preclude the surface from chemical reactions but also a means to induce non-wetting. Therefore, in the case of PVA coating, the corrosion inhibition occurred to the presence of electron donor atoms of nitrogen in its molecular structure. So, the electron lone pair on the nitrogen will coordinate with the metal atoms of the active sites and increase adsorption to hence higher inhibition efficiency, which can be stabilized by the participation of the two adsorption modes, physisorption and chemisorption [23]. PVA/PANI coating presents the advantages of physical barrier protection with PVA and the redox features of PANI [26]. On the other hand, the composite of PVA with GO-COOH, a nanofiller reinforced PVA polymeric coating, acts as an excellent barrier to corrosive solutions and makes its diffusion path available in the tortuous path [27,28]. On the other hand, the carboxylic group on graphene surface nanofillers improve the quality and adherence of the PVA coating, reducing the porosity of the coating matrix and altering the physicochemical properties of the coating–cast iron interface, increasing the anti-corrosion properties [28].

#### 3.2.2. Weight Loss Studies

**Figure 4:**
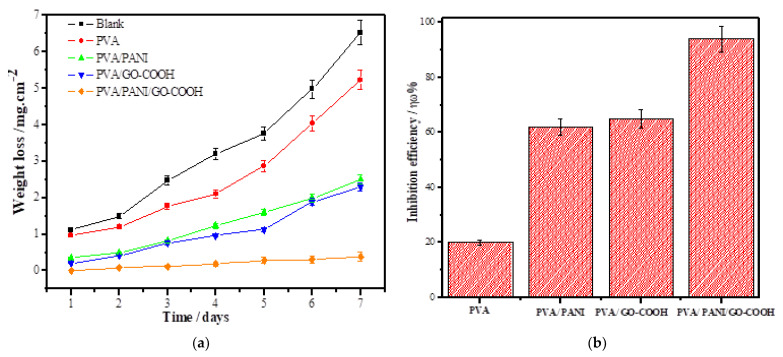
(**a**) Weight loss curves for cast iron in 3.5 wt.% NaCl solution, (**b**) inhibition efficiency, ηω%, in the absence and presence of various coatings.

Figure 4a displays weight loss plots of uncoated and coated samples with PVA, PVA/PANI, PVA/GO-COOH, and PVA/PANI/GO-COOH nanocomposite coating in 3.5 wt.% NaCl aqueous solution. As revealed by the graph, all the coatings showed significant improvement in corrosion resistance. Between all coatings, the PVA/PANI/GO-COOH nanocomposite coating showed a higher resistance in saline solution compared with PVA, PVA/PANI, and PVA/GO-COOH, as displayed by the lower value of weight loss. The data suggested that in the saline environment, the PVA/PANI/GO-COOH nanocomposite coating was more resistant to corrosive ions by 74% more than the PVA coating and resisted the ionic attack. However, PVA coating displayed relatively low resistance to corrosion and provided lower protection, as evident from its comparison with the other coatings.

When PANI comes in contact with moisture or a corrosive medium, the nitrogen atoms in the polymer chain meet with hydrogen atoms and are reduced to the emeraldine (partially oxidized state) or nigeraniline (75% oxidized state) [27], resulting in the chain becoming twisted. This induces porosity in the coating, allowing the solvent molecules to reach other polymer chains beneath the top surface, propagating the reduction reaction and contributing to the growth of a passive oxide layer on the metal surface, protecting them [26]. Still, that probability is minimal due to the hydrogen gas evolution from redox reactions involved in the corrosion process. In the case of GO-COOH present in the composite structure, the approach of protons to the PANI nitrogen atoms is supposed to be hindered because GO-COOH remains essentially inert, permeable only to water molecules, and probably not to the solvated protons or hydronium ions. This suggests that the spread of the PVA/PANI/GO-COOH composite over the cast iron surface is most probably in the form of a network.

#### 3.2.3. Morphological Characterization

**Figure 5:**
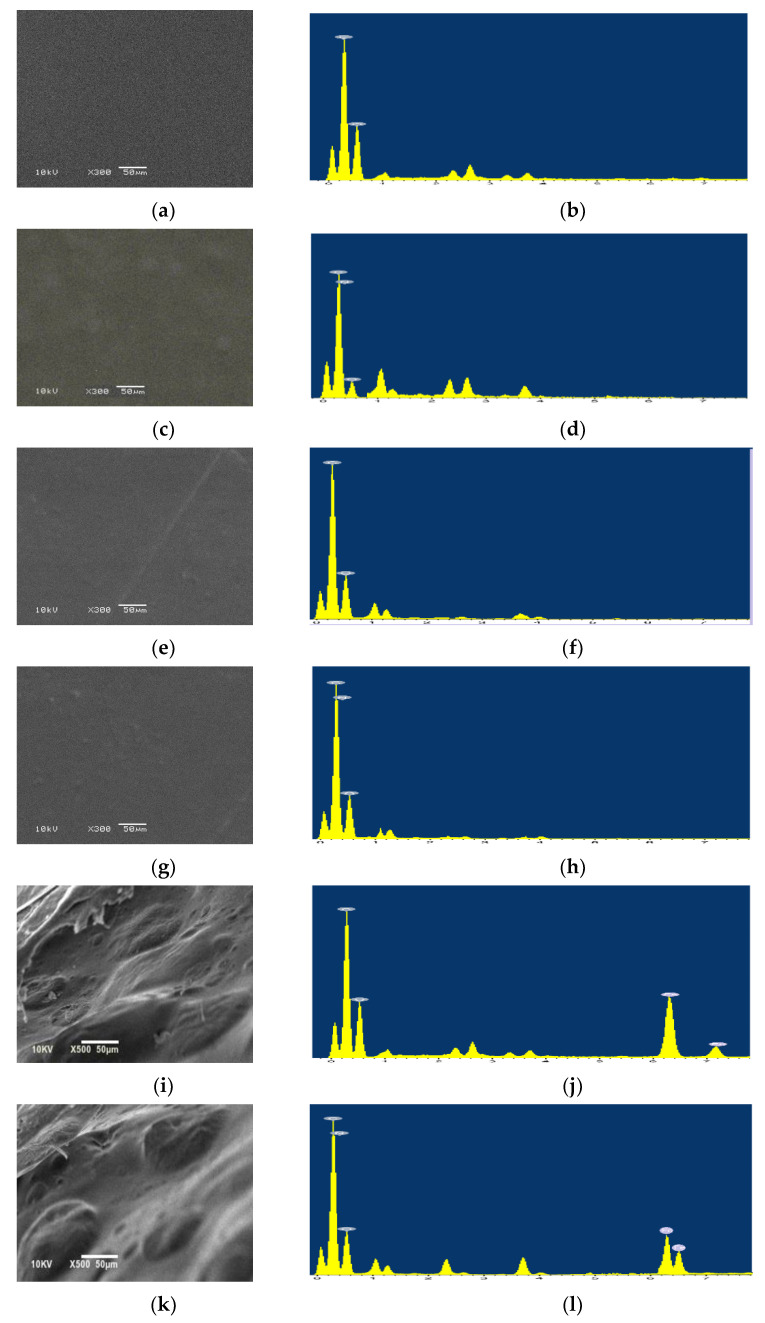

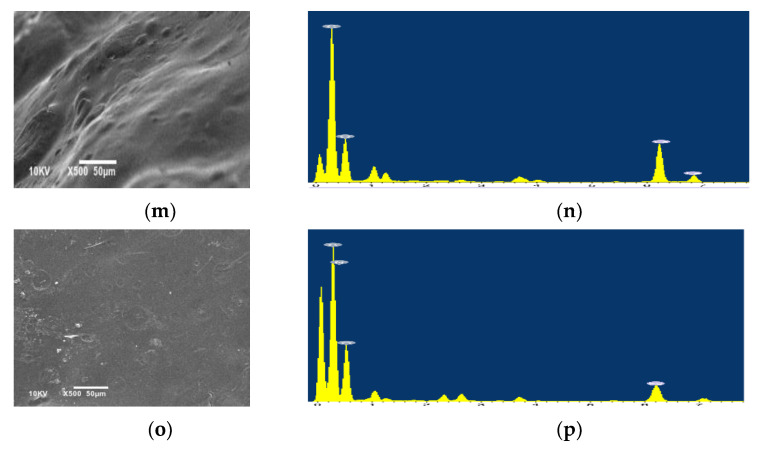
Typical SEM images and EDX spectra of cast iron coupons coated with (**a**,**b**) PVA, (**c**,**d**) PVA/PANI, (**e**,**f**) PVA/GO-COOH, and (**g**,**h**) PVA/PANI/GO-COOH. SEM images and EDX spectra of cast iron coupons coated with (**i**,**j**) PVA, (**k**,**l**) PVA/PANI, (**m**,**n**) PVA/GO-COOH, and (**o**,**p**) PVA/PANI/GO-COOH after 7 days of exposure to 3.5 wt.% NaCl.

Cast iron coupons coated with PVA, PVA/PANI, PVA/GO-COOH, PVA/PANI/GO-COOH show (Figure 5a,c,e,g) a dense and compactly packed adhesive layer. The EDX results (Figure 5b,d,f,h) for coated coupons with PVA, PVA/PANI, PVA/GO-COOH, PVA/PANI/GO-COOH, respectively, show peaks at 0.25 and 0.50 keV due to the presence of C and O. In PVA/PANI and PVA/PANI/GO-COOH coated coupons, an additional peak of N at 0.30 keV was observed, confirming the doping of PAN into PVA matrix. The morphology images of rust coupons coated with PVA, PVA/PANI, PVA/GO-COOH, and PVA/PANI/GO-COOH are shown in Figure 5i,k,m,o. Compared with the large area of damaged coated layer on cast iron coupons with PVA, a circular cake shape corrosion product was observed for PVA/PANI. Less amount was observed for PVA/GO-COOH, and that was not observed for PVA/PANI/GO-COOH coated sample, indicating that the cast iron coupon coated with PVA/PANI/GO-COOH had the weakest degree of corrosion in the different samples, as confirmed by EDX (Figure 5j,l,n,p). 

#### 3.2.4. Electrochemical Characterization

**Figure 6:**
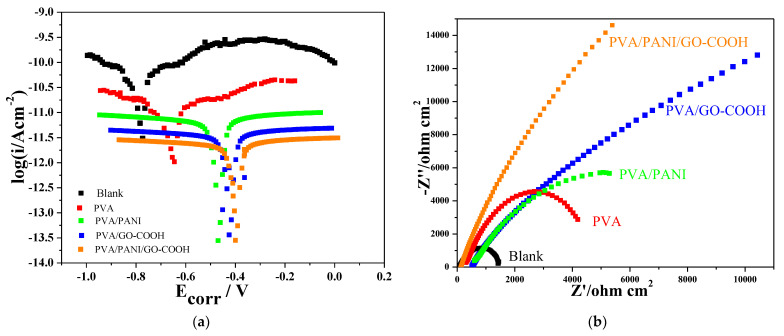
(**a**) Tafel polarization curves and (**b**) Nyquist plot of the coating samples after being immersed in 3.5 wt.% NaCl solution for 7 days.

The corrosion resistance of the uncoated and PVA, PVA/PANI, PVA/GO-COOH, and PVA/PANI/GO-COOH coated samples was evaluated using Tafel analysis. “E-log I” curves for uncoated and coated samples exposed to 3.5 wt.% NaCl solution are represented in Figure 6a. In general, a higher E_corr_ and a lower I_corr_ indicate greater inhibition efficiency [4]. The corrosion potentials of coated samples were positively shifted, and the corrosion current densities were significantly reduced compared to the uncoated cast iron bar. However, PVA/PANI/GO-COOH coated sample showed the highest right shift compared to PVA/PANI and PVA/GO-COOH coatings, and that increase in E_corr_ was an indication of good anodic protection. This may be due to the good compatibility of PVA with PANI and GO-COOH additives and its effect as multiple barriers that effectively promote corrosion resistance by preventing the penetration of corrosive particles to the metal surface.

The Nyquist plot in Figure 6b represents the corrosion behavior of uncoated and coated cast iron samples. All the experiments have been done for uncoated and coated cast iron samples at 35 °C and 0.5 M NaCl solution. It can be observed from the Nyquist plot that the semicircle diameter increases with different coatings, in the order PVA, PVA/PANI, PVA/GO-COOH, and PVA/PANI/GO-COOH. The larger arc radius reflects increasing corrosion resistance, the longer the electrolyte solution takes to corrode the coating, thus enhancing the corrosion resistance. The electrochemical impedance data for PVA/PANI/GO-COOH, which shows the best anti-corrosion performance, was interpreted in terms of the equivalent circuit composed of resistors and capacitors, such as the equivalent circuit shown in Figure S1 of supplementary material, including a solution resistance (R_s_), coating resistance (R_f_), coating capacitance (C_c_), double layer capacitance (C_dl_), and charge transfer resistance (R_ct_). However, the charge transfer resistance value is inversely proportional to the corrosion rate. A high charge transfer resistance value correlates to a low corrosion rate [29], and that was observed with PVA/PANI/GO-COOH coating, which showed the highest R_ct_ value and lowest corrosion rate, confirming the PVA/PANI/GO-COOH coating anti-corrosion effect.

It was concluded that the PVA/PANI/GO-COOH coating was more effective than other coatings. That can be explained by the fact that the PVA coating can isolate and protect the cast iron surface from the external corrosive environment, but during the curing process, some micropores will be formed on the metal surface due to the evaporation of the solvent. Consequently, the oxidizing medium can easily pass through the pores and cause corrosion. However, adding PANI and GO-COOH, which act as filler materials that fill the voids, improves the compactness and provides an extra barrier layer against the corrosive. In addition, PANI dispersed in PVA exerts a unique anti-corrosion effect that can passivate the iron substrate and delay corrosion. At the same time, the conductive properties of GO-COOH prevent the formation of rust.

#### 3.2.5. Optimization of Corrosion Conditions for PVA/PANI/GO-COOH Coating

**Figure 7:**
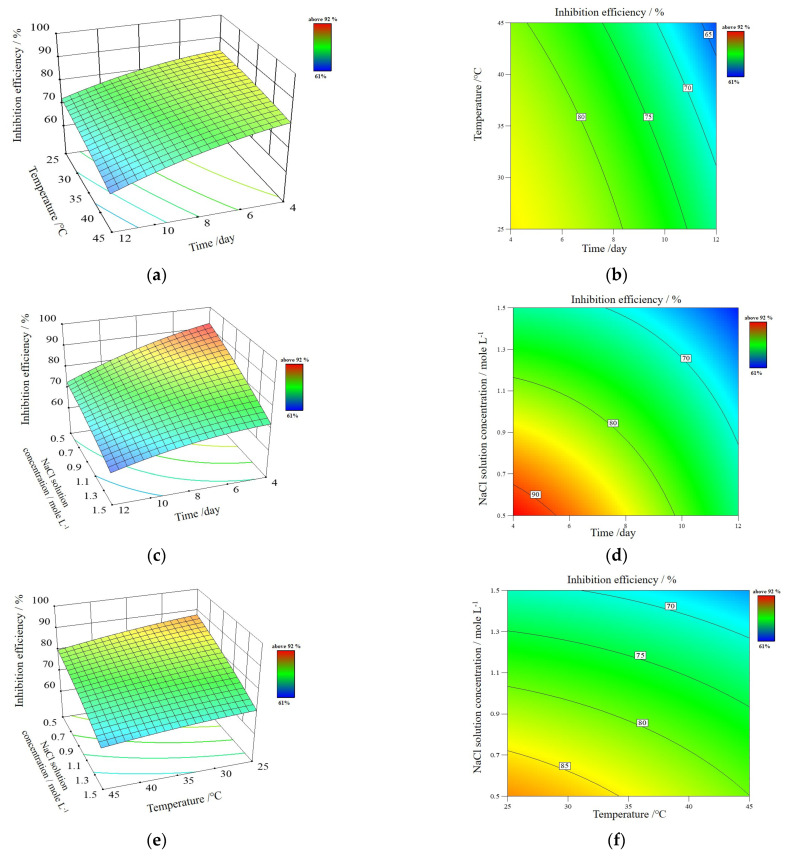
(**a**,**c**,**e**) The 3D surface plots and (**b**,**d**,**f**) 2D surface plots of the inhibition efficiency, ηω%, response of PVA/PANI/GO-COOH nanocomposite coating over the cast iron surface.

The influence of time, temperature, and saline solution concentration on the inhibition efficiency, ηω%, of PVA/PANI/GO-COOH nanocomposite coating over the cast iron surface was investigated and optimized by using a design matrix following the Box-Behnken design [30] with 17 trials, as illustrated in Tables S2 and S3 of supplementary file. The 3D surface plots, as shown in Figure 7, exhibit the findings of the Box–Behnken design, demonstrating the type of interaction between the tested variables and the optimum conditions. The ANOVA analysis of variance is well-known for determining the statistical significance of the quadratic response surface model. As shown in Table S4, the quadratic model is very suitable for a high coefficient of determination R^2^ (0.9606) and low *p*-value (0.0004) of the model, which indicates the model was significant. In addition, *p*-values lower than 0.0500 indicate A, B, and C model terms were significant. However, the model F-value of 18.96 implies the model is significant, and there is only a 0.04% chance that an F-value this large could occur due to noise.

According to the model’s calculations, the numerical relationship between the independent variable and the responses Y (inhibition efficiency) is as follows:

Y= 78 − 7.54A − 3.25B − 7.79C − 2.09A2 − 0.6625B2 − 1.09C2 − 1.25AB + 2.32 AC + 0.75BC(3)

The optimal levels of the three components at the maximum point of the polynomial model were 4 days at 28.7 °C and 0.53 M NaCl solution concentration to obtain maximum inhibition efficiency (about 93%) for PVA/PANI/GO-COOH coating.

## 4. Conclusions

A greater number of pores mean a greater number of corrosive particles pass through the coating, which exposes more surface area of the metal to corrosion reaction. Therefore, the porosity of the coating layer is critical in achieving good corrosion resistance. As a result, the coating with the higher pore resistance in any given environment performed better than the one with the lower coating resistance value. According to the proposed mechanism, PVA/PANI/GO-COOH composite molecules arrange to form an optimized network structure when coated on the cast iron surface and thus provide 94% inhibition efficiency compared to the uncoated sample. The excellent corrosion protection of PVA/PANI/GO-COOH was optimized to 93.3% for 4 days at 0.53 M NaCl solution concentration and 28.7 °C. PVA/PANI/GO-COOH nanocomposite has many advantages such as the eco-friendly component, low cost of production, and large-scale implementation in commercial applications is comparatively convenient.

## Figures and Tables

**Table 1:** Chemical composition of cast iron specimens used.

Element	Composition, wt.%					
	C	Si	Mn	S	P	Fe
	3.17	2.84	0.37	0.12	0.09	93.41

## Data Availability

All data generated or analysed during this study are included in this published article and its Supplementary Information files.

## Supplementary Materials

The following supporting information can be downloaded at: https://www.mdpi.com/article/10.3390/polym14091791/s1, Figure S1: Suggested equivalent circuit for PVA/PANI/GO-COOH coating, Table S1: Level of various independent variables at coded values of response surface methodology experimental design; Table S2: The Box-Behnken design matrix and results for the three variables that influenced on Inhibition efficiency (%) of PVA/PANI/GO-COOH nanocomposite coating; Table S3: The Box-Behnken design matrix and results for the three variables that influenced on Inhibition efficiency (%) of PVA/PANI/GO-COOH nanocomposite coating; Table S4: ANOVA analysis for response function Y (inhibition efficiency (%)).
