# Supplementary material for: Polyvinyl Alcohol/Polyaniline/Carboxylated Graphene Oxide Nanocomposites for Coating Protection of Cast Iron in Simulated Seawater
Source: Polymers (Basel). 2022 Apr 27;14(9):1791. doi: 10.3390/polym14091791 (PMC9099693; doi:10.3390/polym14091791)
Supplement: Supplementary file 1 [file polymers-14-01791-s001.zip › polymers-1645555-supplementary.pdf]

Supplementary Material

Polyvinyl alcohol/Polyaniline/Carboxylated Graphene Oxide Nanocomposites for Coating Protection of Cast Iron in Simulated Seawater

Noha A. Elessawy*[1], Marwa H. Gouda*[2], Mohamed Elnouby[3], Nahla A. Taha[4], M. Elsayed Youssef[1], Diogo M.F. Santos[5]

[1] Computer Based Engineering Applications Dept., Informatics Research Institute IRI, City of Scientific Research & Technological Applications (SRTA-City), 21934 Alexandria, Egypt; nony_essawy@yahoo.com, elsayed168@gmail.com

[2] Polymer Materials Research Department, Advanced Technology and New Materials Research Institute, City of Scientific Research and Technological Applications (SRTA-City), 21934 Alexandria, Egypt; marwagouda777@yahoo.com

[3] Nanomaterials and Composites Research Department, Advanced Technology and New Materials Research Institute, City of Scientific Research and Technological Applications (SRTA-City), 21934 Alexandria, Egypt; mnano2050@yahoo.com

[4] Modelling and Simulation research Department, Advanced Technology and New Materials Research Institute (ATNMRI), City of Scientific Research and Technological Applications (SRTA-City), 21934 Alexandria, Egypt; nahlataha_1982@yahoo.com

[5] Center of Physics and Engineering of Advanced Materials, Laboratory for Physics of Materials and Emerging Technologies, Chemical Engineering Department, Instituto Superior Técnico, Universidade de Lisboa, 1049-001 Lisbon, Portugal; diogosantos@tecnico.ulisboa.pt

* corresponding authors: nony_essawy@yahoo.com; marwagouda777@yahoo.com

Table S1: Level of various independent variables at coded values of response surface methodology experimental design.

Symbol	Independent variables	Coded levels		
		-1	0	1
A	Time / day	4	8	12
B	Temperature / º	25	35	45
C	NaCl solution concentration / mol L^{-1}	0.5	1	1.5

Table S2: The Box-Behnken design matrix and results for the three variables that influenced on Inhibition efficiency (%) of PVA/PANI/GO-COOH nanocomposite coating

Trial	Time (A; day)	Temperature (B; ºC)	NaCl solution concentration (C; mol L^{-1})	Inhibition efficiency (%)	
				Measured	Predicted
**1**	8	45	0.5	79	80.04
**2**	12	35	0.5	76	72.75
**3**	12	45	1	61	63.21
**4**	12	35	1.5	62	61.83
**5**	4	45	1	82	80.79
**6**	4	25	1	87	84.79
**7**	8	35	1	78	78
**8**	8	35	1	78	78
**9**	8	35	1	78	78
**10**	8	25	1.5	72	70.96
**11**	4	35	1.5	69	72.25
**12**	8	35	1	78	78
**13**	4	35	0.5	92.3	92.48
**14**	8	35	1	78	78
**15**	8	25	0.5	86	88.04
**16**	12	25	1	71	72.21
**17**	8	45	1.5	68	65.96

Table S3: ANOVA analysis for response function Y (inhibition efficiency (%)).

Source	Sum of squares	df	Mean square	F-value	p-value
Model	1081.68	9	120.19	18.96	0.0004
A-Time	454.51	1	454.51	71.70	< 0.0001
B-Temperature	84.50	1	84.50	13.33	0.0082
C- NaCl solution concentration	485.16	1	485.16	76.54	< 0.0001
AB	6.25	1	6.25	0.9860	0.3538
AC	21.62	1	21.62	3.41	0.1073
BC	2.25	1	2.25	0.3549	0.5701
A^2	18.35	1	18.35	2.89	0.1327
B^2	1.85	1	1.85	0.2915	0.6060
C^2	4.98	1	4.98	0.7856	0.4049
Residual	44.37	7	6.34		
Lack of Fit	44.37	3	14.79		
Pure Error	0.0000	4	0.0000		
Cor Total	1126.05	16			

**Std. Dev.**	2.52	**R^2**	0.9606
**Mean**	76.19	**Adjusted R^2**	0.9099
**C.V. %**	3.30	**Predicted R^2**	0.3695
		**Adeq. Precision**	15.8726
